# Genetically Engineered Excitable Cardiac Myofibroblasts Coupled to Cardiomyocytes Rescue Normal Propagation and Reduce Arrhythmia Complexity in Heterocellular Monolayers

**DOI:** 10.1371/journal.pone.0055400

**Published:** 2013-02-05

**Authors:** Luqia Hou, Bin Hu, José Jalife

**Affiliations:** 1 Center for Arrhythmia Research, Department of Internal Medicine, University of Michigan, Ann Arbor, Michigan, United States of America; 2 Department of Molecular and Integrative Physiology, University of Michigan, Ann Arbor, Michigan, United States of America; University of Milan, Italy

## Abstract

**Rationale and Objective:**

The use of genetic engineering of unexcitable cells to enable expression of gap junctions and inward rectifier potassium channels has suggested that cell therapies aimed at establishing electrical coupling of unexcitable donor cells to host cardiomyocytes may be arrhythmogenic. Whether similar considerations apply when the donor cells are electrically excitable has not been investigated. Here we tested the hypothesis that adenoviral transfer of genes coding Kir2.1 (I_K1_), Na_V_1.5 (I_Na_) and connexin-43 (Cx43) proteins into neonatal rat ventricular myofibroblasts (NRVF) will convert them into fully excitable cells, rescue rapid conduction velocity (CV) and reduce the incidence of complex reentry arrhythmias in an *in vitro* model.

**Methods and Results:**

We used adenoviral (Ad-) constructs encoding Kir2.1, Na_V_1.5 and Cx43 in NRVF. In single NRVF, Ad-Kir2.1 or Ad-Na_V_1.5 infection enabled us to regulate the densities of I_K1_ and I_Na_, respectively. At varying MOI ratios of 10/10, 5/10 and 5/20, NRVF co-infected with Ad-Kir2.1+ Na_V_1.5 were hyperpolarized and generated action potentials (APs) with upstroke velocities >100 V/s. However, when forming monolayers only the addition of Ad-Cx43 made the excitable NRVF capable of conducting electrical impulses (CV = 20.71±0.79 cm/s). When genetically engineered excitable NRVF overexpressing Kir2.1, Na_V_1.5 and Cx43 were used to replace normal NRVF in heterocellular monolayers that included neonatal rat ventricular myocytes (NRVM), CV was significantly increased (27.59±0.76 cm/s vs. 21.18±0.65 cm/s, p<0.05), reaching values similar to those of pure myocytes monolayers (27.27±0.72 cm/s). Moreover, during reentry, propagation was faster and more organized, with a significantly lower number of wavebreaks in heterocellular monolayers formed by excitable compared with unexcitable NRVF.

**Conclusion:**

Viral transfer of genes coding Kir2.1, Na_V_1.5 and Cx43 to cardiac myofibroblasts endows them with the ability to generate and propagate APs. The results provide proof of concept that cell therapies with excitable donor cells increase safety and reduce arrhythmogenic potential.

## Introduction

Cell therapy, which involves the introduction of new cells into host tissue, is emerging as a novel therapeutic approach in heart disease [1]. Numerous studies have shown that by using bioengineered tissue, including stem and somatic cells, there can be successful modification of the electrophysiological properties of host cardiomyocytes [2], [3], [4]. Further, antiarrhythmic effects of cell therapy have been observed both *in-vitro* and *in-vivo* [5]. However, recent studies suggested that coupling of unexcitable cells, which are commonly used in cell therapy, to cardiomyocytes might yield different functional outcomes that can be arrhythmogenic [6]. Recently, Yankelson et al. showed that when fibroblasts transfected to express the voltage-sensitive potassium channel K_V_1.3 were co-cultured with neonatal rat ventricular myocytes (NRVM) they reduced significantly (68%) the spontaneous beating frequency of the cultures [5]. Subsequently, McSpadden et al. demonstrated that coupling cardiomyocytes with human embryonic kidney (HEK293) cells overexpressing connexin-43 (Cx43) modulates pacemaker activity and slows impulse conduction by as much as 5 times [6]. Miragoli et al. found similar slowing in conduction velocity (CV) in cardiomyocytes monolayers covered with fibroblasts expressing Cx43. Accompanying the reduction of conduction was a significant depolarization in host cardiomyocytes’ resting membrane potential (RMP) from −78 mV to −50 mV [7]. Our previous studies using co-cultures of randomly distributed NRVM and neonatal rat ventricular myofibroblasts (NRVF), which express low levels of Cx43 and repolarizing potassium currents, demonstrated correlations between the proportion of myofibroblasts and the amount of conduction and rotor frequency slowing, the number of wavebreaks, and the degree of fibrillatory conduction [8]. The above studies brought attention to the potential arrhythmogenic consequences of using unexcitable cells in an attempt to regenerate normal cardiac function. To our knowledge, no one has studied the electrophysiological consequences when the donor cells are of cardiac origin and engineered to be fully excitable and capable of generating action potentials (APs).

A crucial condition for any approach used in cell therapy in cardiology is the absence of pro-arrhythmia. This can be achieved when the safety of electrical wave propagation between the donor cells and the host myocardium is preserved by both a high level of excitability and an appropriate degree of intercellular communication at the interface. Therefore, it seems reasonable to assume that, using excitable donor cells fully capable of generating APs as cell therapy would rescue rapid CV and reduce the incidence of complex reentry arrhythmias. To prove this concept we conducted experiments in a heterocellular *in-vitro* model of cardiac impulse propagation. Since NRVF are unexcitable cells lacking sufficient densities of the two essential currents for the generation of ventricular myocyte-like APs (i.e., the inward rectifier current, I_K1_ and the rapid inward sodium current, I_Na_ ) [9], [10], we tested the following two hypotheses: 1. adenoviral transfer of genes coding Kir2.1 (I_K1_) and Na_V_1.5 (I_Na_) proteins converts NRVF into fully excitable cells; 2. adenoviral transfer of genes coding Kir2.1, Na_V_1.5 and Cx43 proteins into NRVF enables them to couple with host cardiomyocytes, rescue rapid CV and reduce the incidence of complex reentry arrhythmias in an *in vitro* model.

## Materials and Methods

### Isolation and Culture of NRVF and NRVM

This study followed guidelines for animal research of the University of Michigan. The protocol was approved by the University Committee on Use and Care of Animals (Approval Number: 09996-2). All surgery was performed after anesthesia by inhalational administration of isofluorane to minimize suffering. Neonatal rats were euthanized by decapitation. Ventricular myofibroblasts and myocytes were isolated and cultured as described by Zlochiver et al. [8]. Briefly, hearts were removed from 1 to 2 day old Sprague-Dawley rats (Charles River Laboratories, MA) and collected in calcium and magnesium free Hanks’ Balanced Salt Solution (HBSS). Ventricles were then isolated, well minced and digested in a solution that contained 0.06% trypsin (Roche Applied Science, IN) and 0.15% pancreatin (Sigma, MO). A two-hour pre-plating period was used to separate early attached NRVF and unattached myocytes. NRVF were cultured in M199 medium (Cambrex, NJ) with 10% fetal bovine serum (FBS) (Cellgro,VA), 20 U/mL penicillin, 20 µg/mL streptomycin; while myocytes were cultured in the same medium with additional 100 µM bromodeoxyuridine (Sigma, MO). Cells were subsequently cultured at 37°C, 5% CO_2_. At passage 3 NRVF were plated sparsely for patch clamp experiments, and at high density to form either pure NRVF monolayers (500,000 NRVF/35 mm dish) or heterocellular monolayers (800,000 NRVM or 80%/35 mm dish; 200,000 NRVF or 20%/35 mm dish) for optical mapping experiments. All NRVF used in this study were passage 3 cardiac myofibroblasts, as demonstrated by α-SMA immunostaining (Figure S1).

### Adenoviral Constructs

We generated adenoviral constructs containing the human Cx43 cDNA sequence (Ad-Cx43), using the AdMax adenoviral vector creation system (Microbix Biosystems, Mississauga, Ontario, Canada). Human Cx43 cDNA was inserted into the pDC315 shuttle vector. The constructed shuttle vector and adeno-genomic plasmid pBHGloxΔE1,3Cre were co-transfected into Microbix 293 cells using the Ca^2+^-phosphate transfection method. Viruses were then purified using ViraBind adenovirus purification kit (Cell Biolabs, San Diego, CA) and the titer was calculated by a plaque forming assay before multicellular preparations were infected with varying multiplicity of infection (MOI). Based on immunostaining 10 MOI was found to be optimal for uniform expression of Cx43 proteins. The adenovirus expressing mouse Kir2.1 (Ad-Kir2.1) was generously provided by Dr. Peter Backx (University Health Network Toronto, Ontario, Canada) for amplification at University of Michigan. The Ad-Na_V_1.5 construct was generously provided by Dr. Silvia Priori (University of Pavia, IRCCS Fondazione Maugeri, Pavia, Italy). 5 and 10 MOI were found optimal for Ad-Kir2.1 infection; 10 and 20 MOI were found optimal for Ad-Na_V_1.5 infection. Viral infections were performed in NRVF at passage 3. All experiments, including patch clamping, fluorescence recovery after photobleaching (FRAP) and optical mapping, were performed at least 48 hours after infection to allow sufficient protein expression.

### Electrophysiology

Voltage clamp experiments were performed in single NRVF infected with Ad-Kir2.1 or Ad-Na_V_1.5 using a MultiClamp 700B amplifier (Axon Instruments, Forest City, CA). I_K1_ recordings were carried out as previously described [11]. The bath solution contained (mM): NaCl 148, NaH_2_PO_4_ 0.4, MgCl_2_ 1, KCl 5.4, CaCl_2_ 1, HEPES 15, pH was adjusted to 7.4 with NaOH. Nifedipine (5 µM) was added to block calcium currents. The pipette solution contained (mM): KCl 148, MgCl_2_ 1, EGTA 5, HEPES 5, creatine 2, ATP 5, phosphocreatine 5, pH was adjusted to 7.2 with KOH. The tip potential was nulled, cell capacitive currents and series resistance were compensated (∼80%) and average cell size was calculated on the basis of cell capacitance (see Figure S2). I_K1_ currents were elicited by 125 ms steps applied in 10 mV increments with a holding potential of 0 mV stepping from −100 mV to +50 mV. BaCl_2_ (1 mM) was used to isolate I_K1_ from background currents. I_Na_ was recorded at room temperature with the pipette filling solution (mM): NaCl 5, CsF 135, EGTA 10, MgATP 5, and HEPES 5 (pH = 7.2). The extracellular solution contained (mM): NaCl 5, MgCl_2_ 1, CaCl_2_ 1, CdCl_2_ 0.1, glucose 11, CsCl 132.5, and HEPES 20 (pH = 7.35). I_Na_ currents were recorded from a holding potential of −160 mV and depolarized to various potentials from −100 to 10 mV in 5 mV increments and 200 ms duration.

APs were recorded in the current clamp configuration using a MultiClamp 700B amplifier from individual NRVF infected with Ad-Kir2.1+ Ad-Na_V_1.5, and Ad-Kir2.1+ Ad-Na_V_1.5+ Ad-Cx43. World Precision Instruments DS8000 stimulator was used to generate square pulses (1–3 ms duration). Extracellular solution was HBSS with Ca^2+^ and Mg^2+^ (Sigma, MO). The pipette solution contained (mM): MgCl_2_ 1, EGTA 5, KCl 150, HEPES 5, Phosphocreatine 5, K_2_ATP 4.5, β-Hydroxybutyric acid 2. All AP recordings were conducted at 37°C.

### Western Blotting

Uninfected and Ad-Cx34 infected NRVF were lysed with RAPA buffer (50 mM Tris-HCl, pH 7.4, 150 mM NaCl, 1% NP-40, 0.25% Na-deoxycholate, 0.1% SDS, 1 mM EDTA) with protease inhibitor cocktail (Sigma, MO). 20 µg proteins were electrophoresed in 4–20% sodium dodecyl sulfate-polyacrylamide gel under reducing condition, and proteins were transferred to polyvinylidene difluoride membrane. The membrane was incubated with rabbit anti-hCx43 antibody (Millipore, MA ) overnight at 4°C after being incubated in blocking buffer (5% fat-free milk in Tris-buffered saline/Tween 20). After washing three times in TBST (100 mM Tris-HCl, pH 7.4, NaCl 145 mM, 0.1% Tween 20), the membrane was then incubated with horseradish peroxidase-conjugated goat anti-rabbit IgG (Jackson ImmunoResearch, PA). Chemiluminescence was developed by the addition of SuperSignal West Pico Chemiluminescent Substrate (Pierce, IL). Signals were detected using radiographic film. For re-probing, the membrane was incubated in Western Blot Stripping Buffer (Pierce, IL) for 30 min at 37°C. Then the membrane was re-blotted with mouse anti-GAPDH antibody and horseradish peroxidase-conjugated goat anti-mouse IgG (Santa Cruz Biotech, CA).

### FRAP Experiments

Uninfected and Ad-Cx43 infected NRVF were loaded with 5-(and-6)-carboxyfluorescein diacetate (5(6)-CFDA) (Invitrogen, NY) (7 µg/mL, 30 min) at 37°C. Regular M199 culture medium was used for the dye loading and during experiments. Dishes were placed in a heating chamber to keep the temperature at 37°C. Single cells were photobleached selectively using a confocal microscope and laser (Nikon A1R). Snapshots of fluorescence images were taken every 15 seconds until 6 minutes after the photobleaching. Target cells that went through photobleaching were labeled with red circles. The cells next to the target cell were labeled with green circles to serve as positive controls. The blue circles were set in the corner areas where no cells were present, in order to measure the background florescence. The recovery of florescence in the target cells were measured and normalized to the florescence right before the photobleaching. The time course of recovery of 5(6)-CFDA in target cells was fit with an exponential curve using GraphPad Prism 5.

### Optical Mapping

Monolayers were placed on a heating stage (37°C) and superfused constantly with non-carbonated HBSS (Sigma, MO) containing (mM): CaCl_2_1.6, KCl 5.4, MgSO_4_ 0.8, KH_2_PO_4_ 0.4, NaHCO_3_ 4.2, NaCl 136.9, Na_2_HPO_4_ 0.3, D-Glucose 5.5, and HEPES 10; pH 7.4. A high-resolution CCD camera (80×80 pixels, SciMeasure Analytical Systems Inc, GA) was used to record electrical wave propagation after staining the dishes with 40 µM/L di-8-ANEPPS (Molecular Probes, NY) for 15 minutes. Monolayers were paced at 2X threshold through a bipolar electrode. Incremental pacing started at 1 Hz (5 ms duration), until loss of 1∶1 capture or induction of sustained reentry. 5-s movies were obtained at 200 frames/second and subsequently analyzed offline.

### Analyses of Optical Movies

All movies were subjected to background fluorescence subtraction and filtered using spatial (3×3 pixels) and temporal (7 pixels) conical convolution filters. Phase maps were generated during sustained reentry to measure rotation frequency and phase singularities (PS) [12]. Dominant frequency (DF) maps and phase maps were constructed as described previously [13]. DF and PS were calculated and averaged from each frame. For CV measurements, we conducted ensemble averaging for each pixel for over 5 or more propagating wave fronts following each stimulus. After generating an ensemble averaged movie the activation time distributions (50% of upstroke) for the spatial regions of 5×5 pixels were fitted with the plane, and gradients of activation times g_x_ and g_y_ were calculated for each plane along the *x* and *y* axes, respectively. We calculated the magnitude of the local CV for each pixel as (g_x_^2^+ g_y_^2^)^−1/2^. We then plotted mean values and standard deviations for CV.

### Statistical Analyses

Statistical analyses were performed using Origin 6.0 and Prism 5. Two-way ANOVA with Bonferroni post-tests was used for patch clamping and FRAP results. Analyses of rotation frequency and PS were performed using one-way ANOVA with Tukey’s multiple comparison test. A Student’s *t-test* with Welch-correction was used to analyze the average CV. Data are expressed as mean±SEM. p<0.05 was considered to be significant.

## Results

### Kir2.1 and Na_V_1.5 Overexpression in NRVF

**Figure 1:**
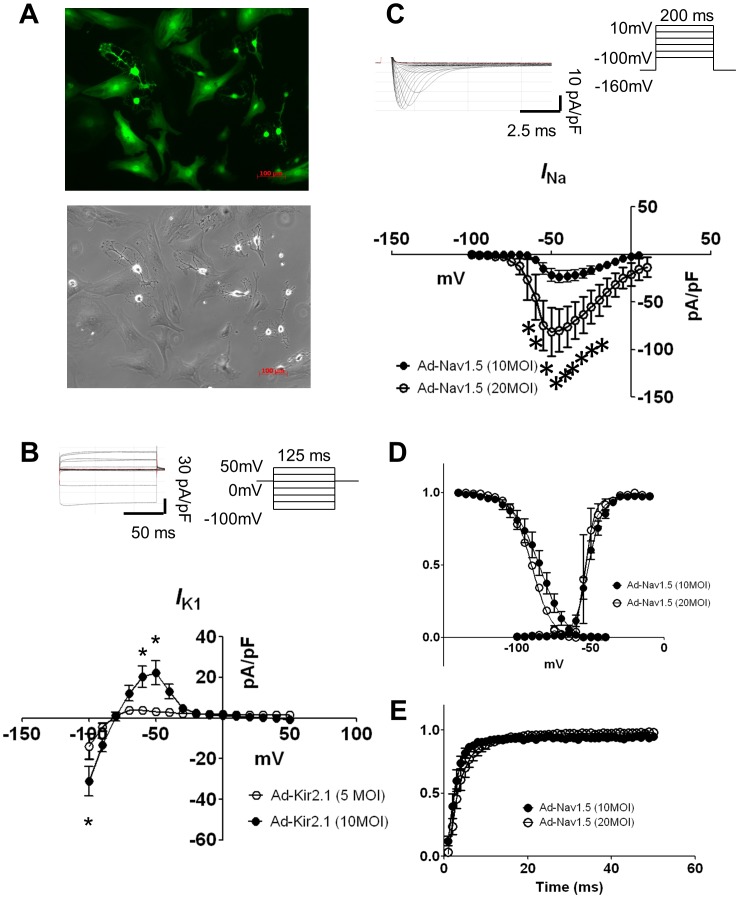
Adenoviral expressions of Kir2.1 and Na_V_1.5 proteins in NRVF after 48 hours of infection. **A.** GFP-tagged Kir2.1 channel expression. Left: fluorescent micrograph of a passage 3 NRVF 48 h after Ad-Kir2.1 infection. Right: corresponding phase contrast micrograph. Scale bar = 100 µm. **B.** Voltage clamp protocol (top right) and representative example of currents (top left) from Ad-Kir2.1 infected NRVF; and I–V relationship (bottom) of the BaCl_2_ sensitive currents normalized to cell capacitance in NRVF infected with Ad-Kir2.1 at 5 MOI (open circles) and 10 MOI (filled circles). **C.** Voltage clamp protocol (top right) and representative example of currents from Ad-Na_V_1.5 (top left) infected NRVF; and I–V relationship (bottom) of I_Na_ normalized to cell capacitance in NRVF infected with Ad-Na_V_1.5 at 10 MOI (filled circles) and 20 MOI (open circles). **D.** Normalized activation and inactivation curves of I_Na_. **E.** Normalized recovery from inactivation curve of I_Na_. The curves in D and E are not different statistically. *: p<0.05.

To our knowledge, constitutive expression of Kir2.1 has not been described for NRVF. At 5 MOI and 10 MOI of adenoviral Kir2.1 expression, we consistently observed uniform GFP expression in single NRVF, as illustrated in Figure 1A. I_K1_ densities were determined by voltage clamp experiments. Figure 1B shows the voltage clamp protocol on the top right (see Methods) and representative Ba^2+^ sensitive I_K1_ traces on the top left. Both inward and outward I_K1_ increased when MOI was increased from 5 to 10 MOI. I_K1_ density vs voltage (IV) plots are shown on the bottom of Figure 1B. Both the peak inward and outward currents were significantly increased in 10 MOI (n = 4) Ad-Kir2.1 infected NRVF comparing to 5 MOI (n = 7) infected NRVF (−100 mV: −31.08±7.28 pA/pF vs. −13.83±6.12 pA/pF, p<0.01; −50 mV: 22.22 V±5.92 pA/pF vs. 3.34±0.92 pA/pF, p<0.01). Although the peak inward and outward currents in 5 MOI infected NRVF were still double the amount of what reported previously in NRVM [14], we chose this MOI for the following monolayer experiments in order to keep a homogenous infection through a dish containing 500, 000 NRVF.

Na_V_1.5 expression was achieved by infecting NRVF with Ad-Na_V_1.5 at 10 MOI and 20 MOI. Average I_Na_ IV plots are presented in Figure 1C, with representative traces depicted on the top left and voltage-clamp protocol on the top right. Peak I_Na_ increased significantly when the MOI was increased from 10 to 20 MOI (−21.64±6.64 pA/pF, n = 8 vs. −81.27±25.41 pA/pF, n = 4, p<0.001), reaching similar values as in native NRVM [15]. However, normalized voltage dependence of activation and inactivation, and recovery from inactivation plots showed no difference between the two groups (Figure 1D and E).

### Kir2.1 and Na_V_1.5 Functional Expression Enabled AP Generation in Single NRVF

**Figure 2:**
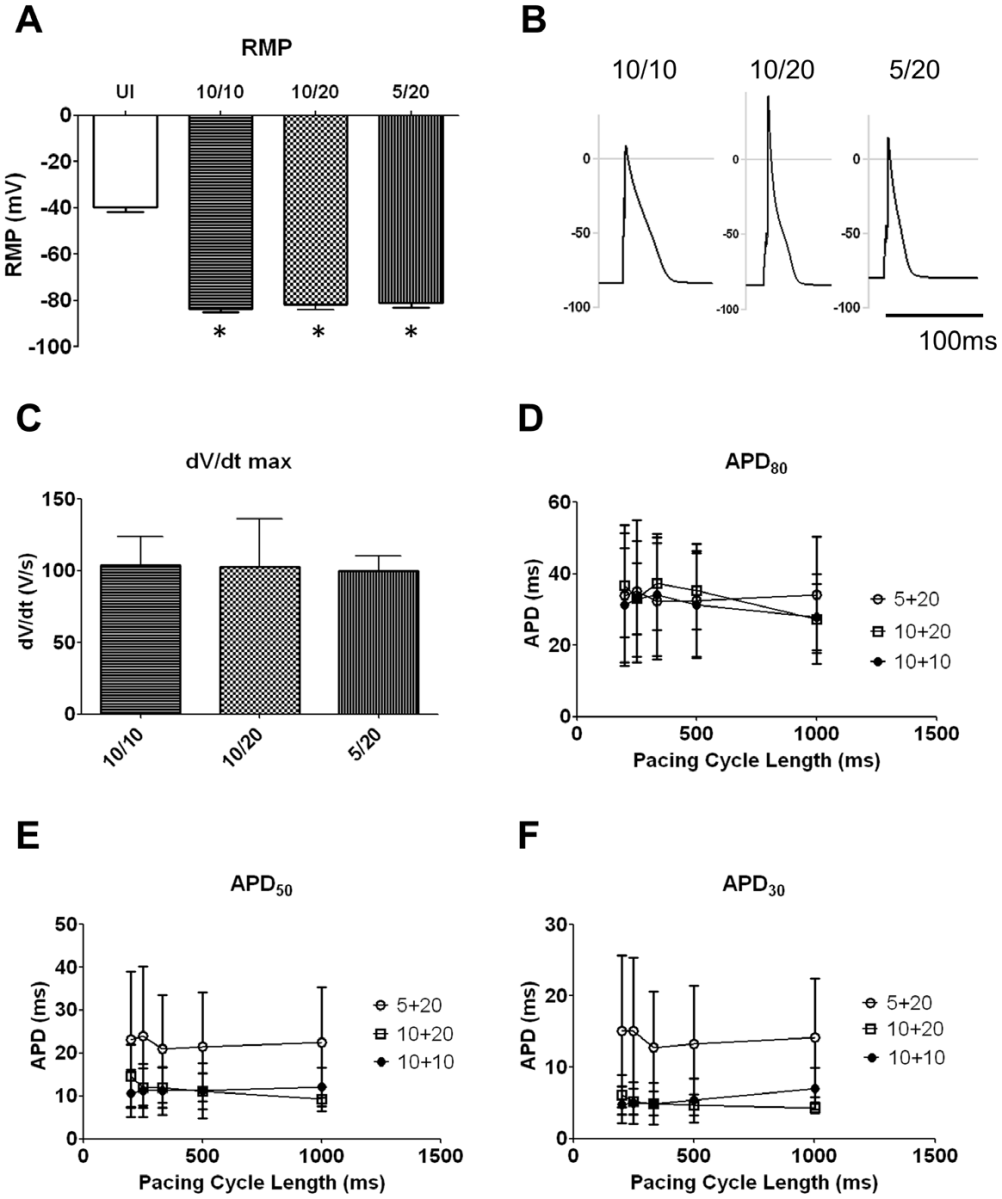
Characterizations of action potentials in NRVF co-infected with Ad-Kir2.1 and Ad-Na_V_1.5 (K/Na). A. Resting membrane potentials from uninfected and co-infected NRVF (K/Na: 10/10, 10/20 and 5/20 MOI). **B.** Representative action potential recordings from NRVF infected with K/Na at 10/10, 10/20 and 5/20 MOI. **C.** Maximal upstroke velocity measured from co-infected NRVF (10/10, 10/20 and 5/20 MOI, NS). **D- F.** Action potential durations (APD)_80_, APD_50_ and APD_30_ in co-infected NRVF (10/10, filled circles; 10/20, open square; and 5/20, open circles; NS).

As shown in the previous section, NRVF infected with Ad-Kir2.1 plus Ad-Na_V_1.5 demonstrated robust I_Na_ and I_K1_ currents, which suggested that genetically engineered myofibroblasts had become excitable and were capable of generating APs. Thus, we co-infected three groups of NRVF with Ad-Kir2.1 and Ad-Na_V_1.5 (K/Na) at different MOI ratios: 10/10 (n = 7), 10/20 (n = 8), and 5/20 MOI (n = 7), in an effort to optimize the conditions for excitability. The RMP was hyperpolarized in all three K/Na groups with respect to control, as illustrated in Figure 2A (p<0.05 vs. UI). In addition, as shown in Figure 2B, unlike control cells, current-clamp experiments enabled AP recordings from all three groups of co-infected NRVF. Quantification of the AP maximum upstroke velocity (dV/dtmax) in each of the three K/Na groups yielded values of ∼100 V/s on average (Figure 2C). As summarized in Figure 2D–F, AP shapes were variable but in all cases repolarization phase showed a characteristic plateau-like morphology, owing to the strong inward rectification of I_K1_ at voltages between −50 and 0 mV (see Figure 1B) [16]. However, AP durations (APDs) measured at 30, 50 and 80% repolarization were relatively brief in all three groups, with no statistically significant difference among groups.

### Cx43 Overexpression Increased Coupling between Neighboring NRVF

**Figure 3:**
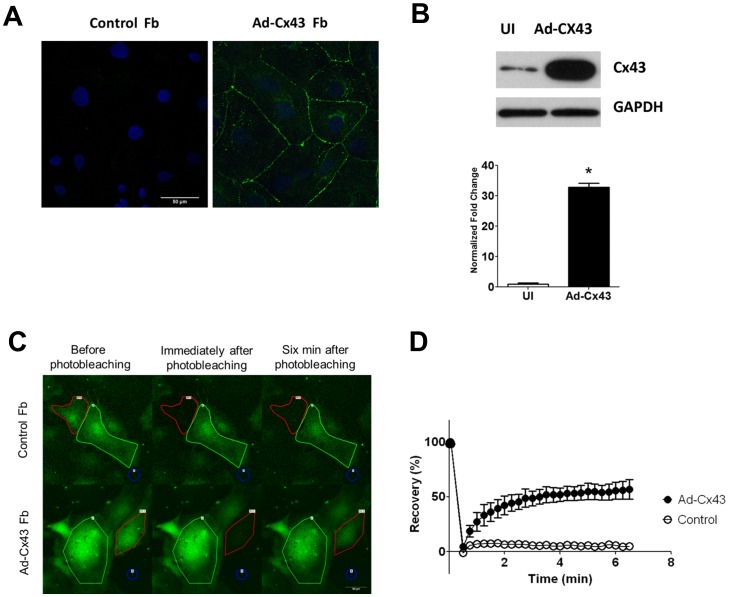
Adenoviral expressions of Cx43 in NRVF after 48 hours of infection. **A.** Immunostaining of control and Ad-Cx43 infected NRVF using an antibody for Cx43 (green) showed increased expression of Cx43 on cell membrane. Scale bar = 50 µm **B.** Western blot showed a 30-fold increase in the total amount of Cx43 proteins in Ad-Cx43 infected myofibroblasts compared with control uninfected NRVF. **C.** FRAP experiments showed strong functional coupling between Ad-Cx43 infected NRVF. Red circles indicated the target cells that were photobleached. Green circles showed the possible donor cells next to target cells. Blue and yellow circles were used to evaluate the background fluorescence. **D.** Quantification of the florescence recovery within six minutes after photobleaching in control (open circles) and Ad-Cx43 infected (filled circles) NRVF.

Previous studies suggested that Cx43 was expressed in cultured cardiac myofibroblasts [8]. Thus we used immunohistochemistry in confluent NRVF monolayers to examine the level of Cx43 expression and determine whether APs would propagate across the monolayer. As shown by the top panels of Figure 3A, Cx43 was nearly undetectable in uninfected NRVF monolayers. As expected, despite the fact that these monolayers expressed robust levels of both Kir2.1 and Na_V_1.5, they failed to propagate APs. Therefore, we constructed an adenovirus encoding human Cx43 sequence (Ad-Cx43) to overexpress Cx43 proteins. The right panel of Figure 3A illustrates the outcome of using the Ad-Cx43 construct. Cx43 was immunolocalized throughout on the cell membrane and also the nucleus area of myofibroblasts after 48 hours infection. Western blot results indicated that there is a more than 30 fold increase in total Cx43 expressions after infection (Figure 3B).

We used FRAP as a functional assay to determine whether overexpression of Cx43 increased cell-to-cell communication in the NRVF monolayer. Once loaded, the CFDA dye was retained in the cells because they were hydrolyzed by cytoplasmic esterases into carboxyfluorescein. However, they were able to permeate through functional gap junctions due to their low molecular weights (376 Da) [17]. Therefore, after photobleaching, unbleached dye molecules would redistribute to the target cell through gap junctions. Figure 3C shows representative recordings in a control NRVF monolayer and an Ad-Cx43 infected NRVF monolayer, before and after the photobleaching. The target cells for photobleaching were outlined in red in both cases. After 6 minutes, fluorescence barely recovered in uninfected NRVF (n = 4), suggesting weak intercellular coupling among normal NRVF. In contrast, Ad-Cx43 infected (n = 3) NRVF showed a significant florescence recovery (>60%) after 6 minutes suggesting the florescent dye was able to travel through the overexpressed Cx43 from the neighboring NRVF. Normalized florescence recovery plots were summarized in Figure 3D. The exponential curves are presented in Figure S3.

### Kir2.1/Na_V_1.5/Cx43 Overexpression Enables fast AP Propagation in NRVF Monolayers

**Figure 4:**
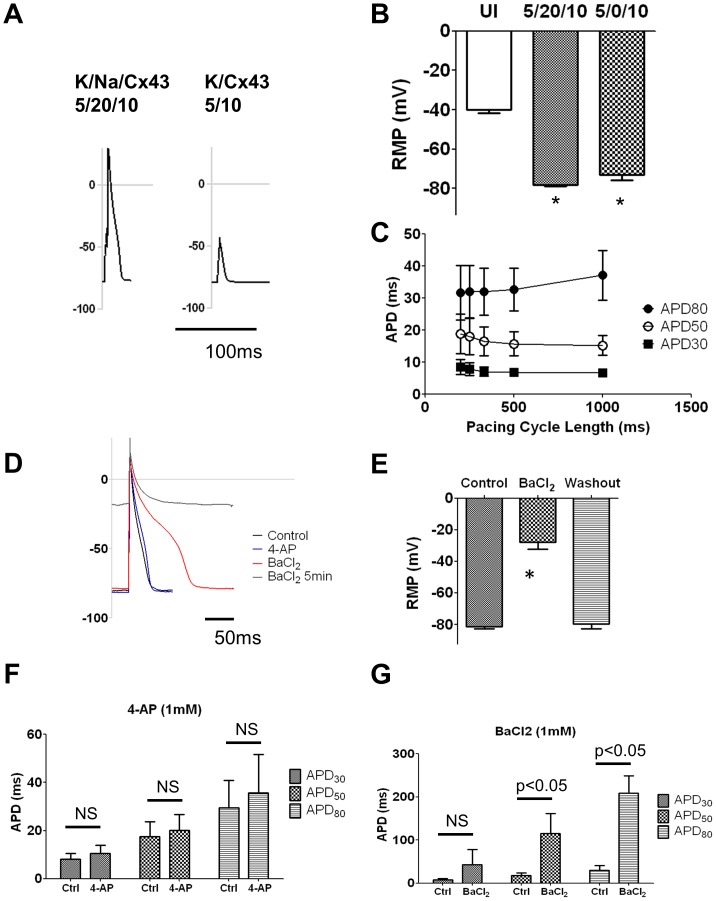
Characterizations of action potentials in NRVF infected with Ad-Kir2.1, Ad-Na_V_1.5 and Ad-Cx43. **A.** Action potentials were recorded from triple infected (K/Na/Cx43, 5/20/10 MOI) NRVF (left) but not in K/Cx43 (5/10 MOI) infected NRVF (right). **B.** Resting membrane potentials in uninfected, K/Na/Cx43 (5/20/10 MOI) infected and K/Cx43 (5/10 MOI) infected NRVF. **C.** APD_80_, APD_50_ and APD_30_ measured at different pacing cycle length. **D.** Representative action potential traces shows that resting membrane potentials and APDs are sensitive to BaCl_2_ (1 mM) but not 4-AP (1 mM). **E.** Resting membrane potentials measured before and after BaCl_2_ (1 mM) perfusion, and after washout in K/Na/Cx43 NRVF. **F.** Quantification of APD_30_, APD_50_ and APD_80_ before and after 4-AP perfusion. **G.** Quantification of APD_30_, APD_50_ and APD_80_ before and after BaCl_2_ perfusion. *: p<0.05.

Using 5 MOI Ad-Kir2.1 and 20 MOI Ad-Na_V_1.5, I_K1_ and I_Na_ current densities in NRVF reached similar levels as the corresponding values in NRVM (see Figures 1 and 2) [14], [15]. Therefore we chose this ratio in double infection experiments to determine whether monolayers formed by excitable NRVF overexpressing Cx43 can propagate APs at velocities equivalent to those of myocytes. In triple infected NRVF (K/Na/Cx43 at 5/20/10 MOI), we observed a significant reduction in input membrane resistance (106.85±51.07 MΩ, n = 6 vs. 301.95±75.25 MΩ, n = 4; p<0.05) without significant change in the size of the cells (70.54±16.37 pF, n = 6, vs. 117.74±33.09 pF, n = 4, NS) (Figure S2). We further confirmed that the triple infected NRVF were electrically excitable and that their morphology did not change after infection during patch clamp experiments (Figure S4). Figure 4A illustrated representative AP recordings. A K/Na/Cx43 NRVF (Figure 4A left) generated large amplitude APs with rapid upstroke velocities, ∼ 30 mV overshoot and ∼ 40 ms duration similar to the morphology of the K/Na NRVF (Figure 2B). On the other hand, in the absence of Na_V_1.5, a K/Cx43 NRVF was unable to generate APs (Figure 4A right) despite having a RMP of ∼ −72 mV (Figure 4B). Thus the endogenous sodium current [18] was insufficient to depolarize the NRVF to threshold. Figure 4B shows that the RMP values for both K/Na/Cx43 (n = 6) and K/Cx43 (n = 5) NRVF were similar to each other but significantly more negative than uninfected (n = 3) NRVF. Figure 4C summarizes the values for APD_30_, APD_50_ and APD_80_ measured at pacing cycle lengths between 200 and 1000 ms. The average APD_80_ at 1 Hz pacing was 37.13±7.78 ms in K/Na/Cx43 NRVF (n = 4).

To determine whether the overexpressed I_K1_ rather than the endogenous delayed rectifier potassium currents [18] was responsible for the AP shape during repolarization, we compared the effects of BaCl_2_ (1 mM) versus 4-AP (1 mM) in single excitable NRVF. As shown in Figure 4D, while 4-AP (blue trace) did not affect either the AP shape or the RMP in any significant way, BaCl_2_ (red trace) initially prolonged APD and subsequently depolarized the RMP of the K/Na/Cx43 cells to the same level as the UI NRVF. The effect of BaCl_2_ was fully reversed after a 5 min washout period (Figure 4E, n = 4). Figure 4F and G summarized the effect of 4-AP (1 mM, n = 5) or BaCl_2_ (1 mM, n = 4) on APD_30_, APD_50_ and APD_80_. While 4-AP did not modify APDs on all levels, BaCl_2_ significantly prolonged both APD_50_ and APD_80_ (p<0.05 vs. control).

**Figure 5:**
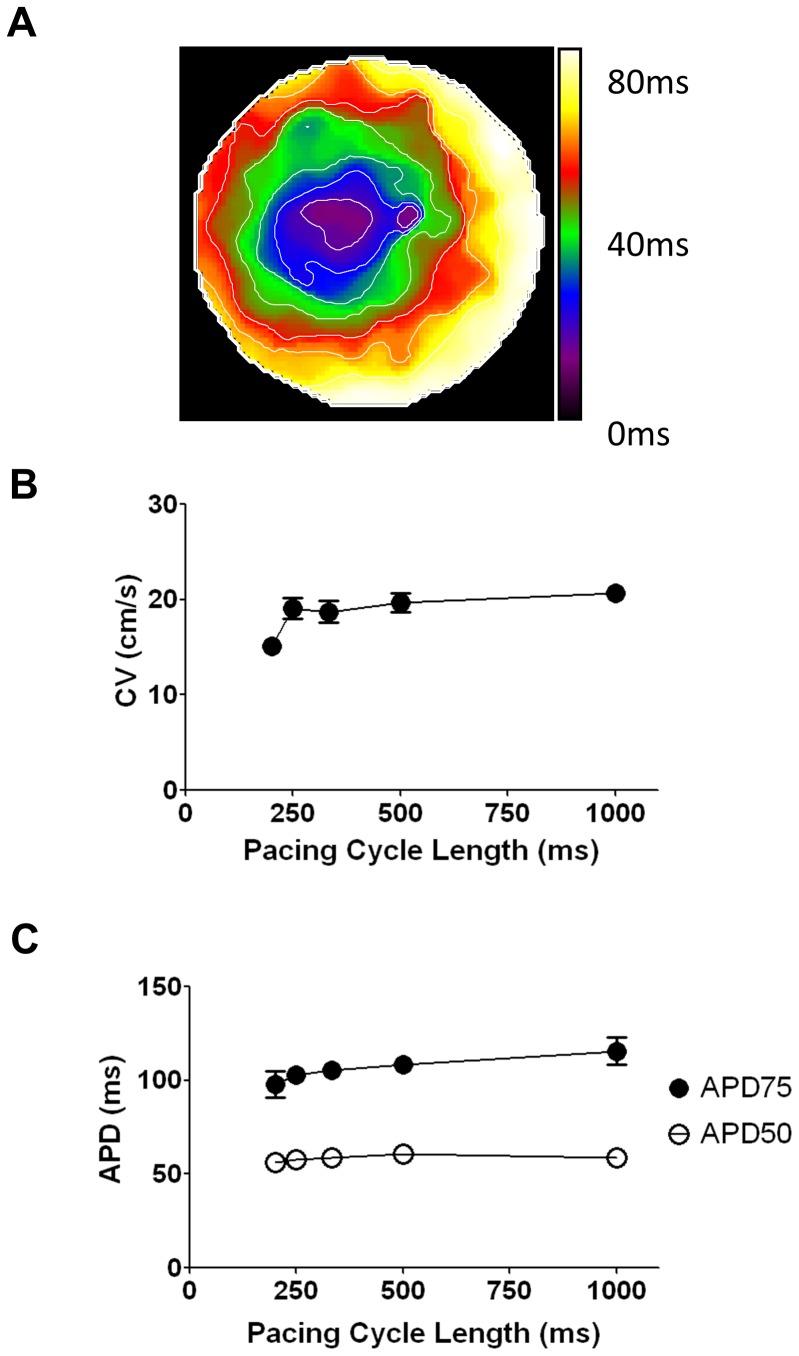
Action potential propagation in 2D monolayer of K/Na/Cx43 NRVF. **A.** Representative activation map of action potential propagation (1Hz pacing) in K/Na/Cx43 NRVF monolayer. Scale bar = 10 mm. **B.** Conduction velocity measured at different pacing cycle lengths from 1000 ms to 200 ms (n = 7). **C.** APD_75_ and APD_50_ measured at pacing cycle lengths from 1000 ms to 200 ms (n = 7).

In high resolution optical mapping experiments, K/Na/Cx43 NRVF plated as 2 dimensional (2D) monolayers, propagated APs at velocities (>20 cm/s) that were comparable to those of NRVM monolayers. Figure 5A is an activation map generated from an excitable NRVF monolayer paced at 1 Hz. Average CVs were quantified and plotted at varying pacing cycle lengths in Figure 5B. The average CV at 1 Hz was 20.71±0.79 cm/s (n = 7). The optical APD_75_ and APD_50_ were also measured and summarized in Figure 5C.

### Kir2.1/Na_V_1.5/Cx43 NRVF Rescue CV in Heterocellular Monolayers

**Figure 6:**
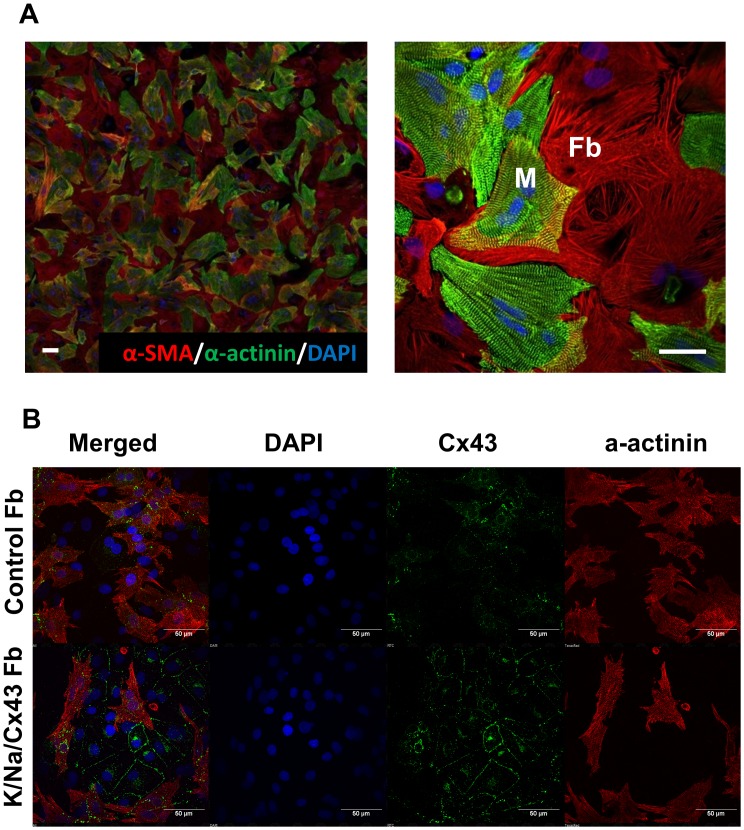
Interaction of NRVF and NRVM in an *in vitro* heterocellular monolayer model. A. Co-immunostaining of NRVM/NRVF co-culture using an antibody for α-SMA (red) and an antibody for α-actinin (green). Scale bar = 10 µm. **B.** Characterization of Cx43 expression between NRVF and NRVM in heterocellular monolayers (uninfected NRVF vs. K/Na/Cx43 NRVF). Scale bar = 50 µm.

Previously, we investigated the effects of myocyte-myofibroblast interactions on wave propagation dynamics in monolayers of co-cultured NRVM and NRVF [8]. We demonstrated CV diminished with larger myofibroblast/myocyte area ratios. To determine whether NRVF made excitable by K/Na/Cx43 overexpression can serve to rescue propagation, we generated heterocellular monolayers consisting of randomly distributed mixtures of NRVM and excitable NRVF, and compared their properties with those of heterocellular monolayers containing unexcitable (i.e., uninfected) NRVF at the same myofibroblast/myocyte ratio (80% myocytes/20% myofibroblasts). Figure 6A illustrates the random distribution of the two cell types at two different levels of magnification. In Figure 6B, we compare representative uninfected (control) and K/Na/Cx43 monolayers immunostained for α-actinin, Cx43 and DAPI. The expression of Cx43 at myocyte-to-myofibroblast contacts was significantly increased in excitable NRVF monolayers comparing to control (see also Figure S5).

**Figure 7:**
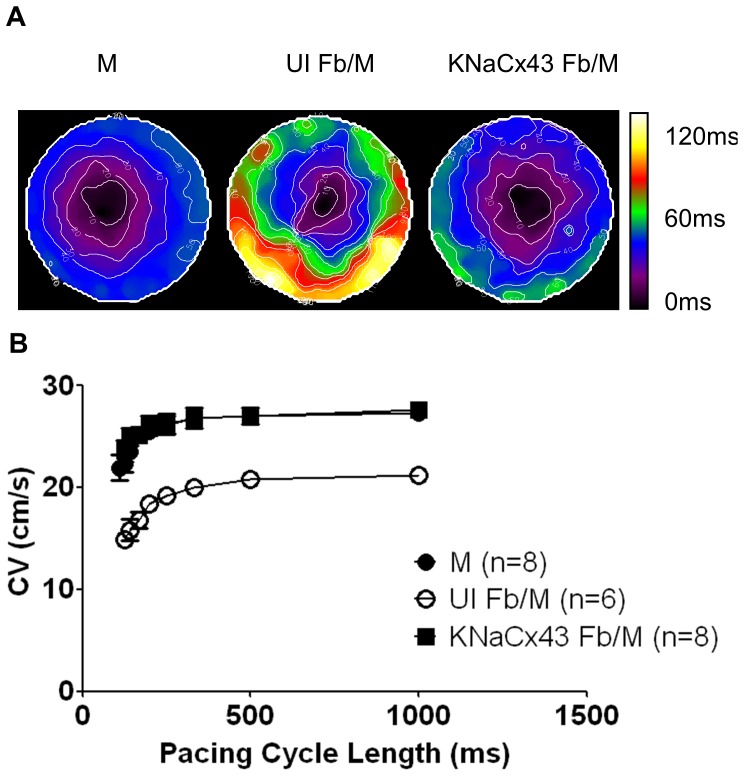
K/Na/Cx43 NRVF rescued normal conduction velocity. A. Activation maps from monolayers of myocytes only (M, left), uninfected NRVF/NRVM co-culture (UI Fb/M, middle), and K/Na/Cx43 NRVF/NRVM co-culture (K/Na/Cx43 Fb/M, right). **B.** Quantification of conduction velocities at varies pacing cycle lengths in monolayers of myocytes (filled circles), UI Fb/M (open circle), and K/Na/Cx43 Fb/M (filled square).

Optical mapping was then performed in three groups of monolayers: pure myocyte (M) monolayers acting as control, heterocellular monolayers containing uninfected myofibroblasts and myocytes (UI Fb/M), and heterocellular monolayers containing excitable K/Na/Cx43 myofibroblasts and myocytes (K/Na/Cx43 Fb/M) at the same ratio (80% myocytes; 20% myofibroblasts). Figure 7A shows representative activation maps for each of the three groups of monolayers obtained during pacing at 1 Hz. Clearly conduction in the UI Fb/M monolayer (middle) was very slow compared to control (left). However, when the UI NRVF were replaced by the same number of excitable myofibroblasts (right), the activation time was restored to the control level. Composite results from 6–8 monolayers in each group are shown in Figure 7B. Quantification of CV at varying pacing cycle lengths demonstrated that the propagation velocities were significantly reduced in the UI Fb/M monolayers compared to control (21.18±0.65 cm/s, n = 6 vs. 27.27±0.72 cm/s, n = 8, p<0.05); however, excitable NRVF (K/Na/Cx43 Fb/M) fully restored fast CV (27.59±0.76 cm/s, n = 8, NS vs. M).

### Kir2.1/Na_V_1.5/Cx43 NRVF Rescued Simple Reentry Patterns

**Figure 8:**
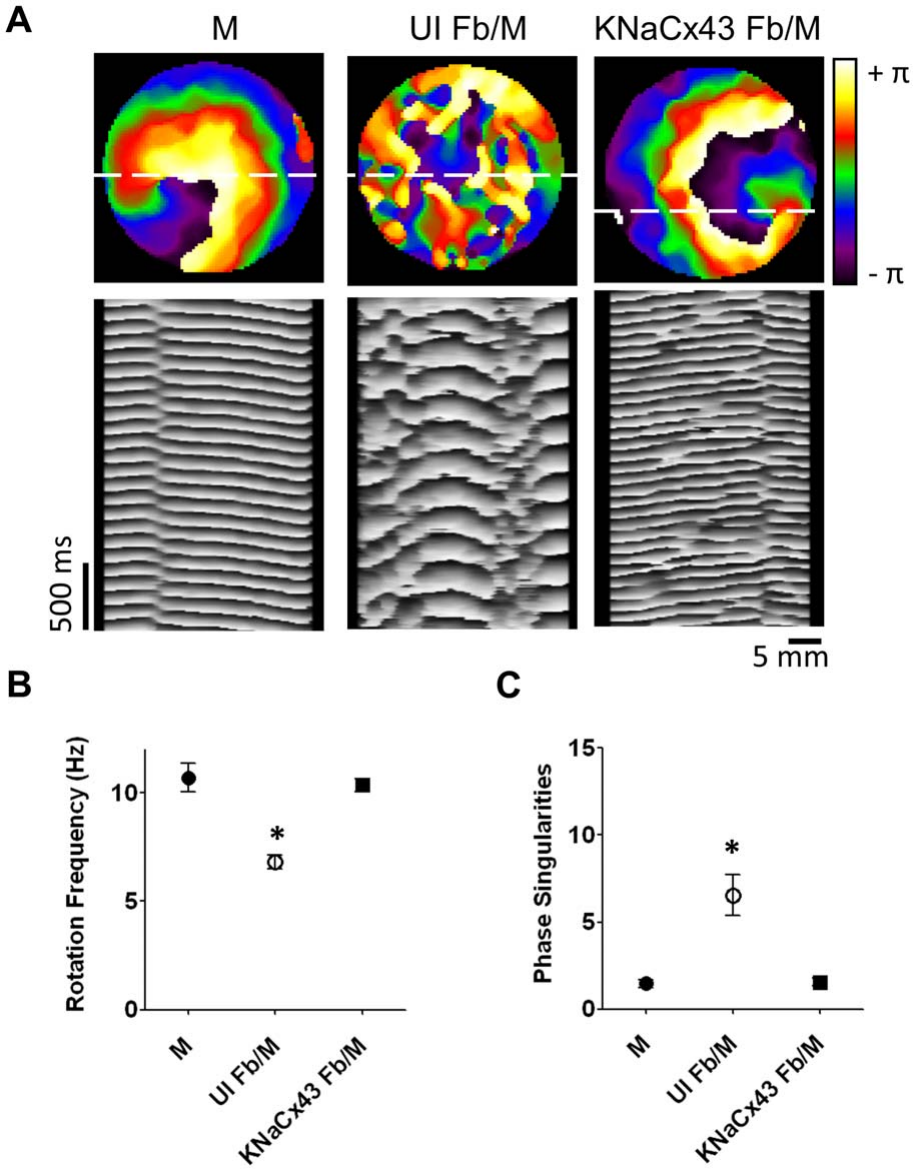
K/Na/Cx43 NRVF increased reentry frequency and reduced wavebreaks. A. Snapshots (top) and Time-Space Plot (bottom) from representative optical mapping movies in monolayers of myocytes (left), UI Fb/M (middle), and K/Na/Cx43 Fb/M (right). **B.** Quantification of rotation frequencies in monolayers of myocytes (filled circle), UI Fb/M (open circle), and K/Na/Cx43 Fb/M (filled square). **C.** Quantification of phase singularities in monolayers of myocytes (filled circle), UI Fb/M (open circle), and K/Na/Cx43 Fb/M (filled square).

As demonstrated previously, increasing the proportion of myofibroblasts in the monolayer reduced both CV and the frequency of reentry but increased the complexity of propagation during reentry resulting in fractionation, wavebreaks and increased number of wavelets [8]. Here we investigated whether the presence of excitable K/Na/Cx43 myofibroblasts prevents such effects and restores the well-organized reentry patterns that are normally observed in monolayers containing >95% myocytes [8], [13]. Thus, we compared the dynamics of reentry in K/Na/Cx43 Fb/M monolayers with those of UI Fb/M and pure M monolayers. Sustained functional reentry could be observed in all three groups whether spontaneously or pacing induced. The top of Figure 8A shows phase maps obtained during reentry in each of the three groups. In these maps, each color indicates one phase of the AP, and the convergence of all colors at the center of rotation is defined as a phase singularity (PS) [19]. Under each phase map is a corresponding time-space plot (TSP) constructed along the horizontal dashed line [20], to quantify the temporal evolution of the electrical activity in each monolayer. Consistent with our previous findings [8], the spatio-temporal characteristics of reentry were significantly more complex in the UI Fb/M monolayers (center) than in the M monolayer (left) or the K/Na/Cx43 Fb/M monolayer. Clearly, the number of PS and the complexity of wave propagation were appreciably greater, but the frequency of reentry was significantly lower in the UI Fb/M monolayer than in the other two. These data are consistent with the CV measurements plotted in Figure 7B.

Figure 8B summarized the rotation frequencies from the three groups of monolayers. Clearly the UI Fb/M monolayers had slower reentry frequency than control myocyte monolayers (6.81±0.32 Hz, n = 10, vs 10.71±0.65 Hz, n = 6, p<0.05). However, by increasing both excitability and intercellular conductivity, K/Na/Cx43 overexpression in the Fb/M monolayer restored the rotation frequency (10.35±0.28 Hz, n = 11, NS vs. M) to control levels. In Figure 8C the maximal number of PS was counted in each frame of a 5-s movie and the average was calculated and then plotted for each group [8]. As expected, the UI Fb/M monolayers had a larger number of wavebreaks and PS (6.54±1.16, n = 10, vs. 1.47±0.23, n = 6, p<0.05) underlying increased complexity of AP propagation. Conversely, the number of PS per frame in excitable K/Na/Cx43 Fb/M monolayers was similar to the control monolayer (1.54±0.23, n = 11 vs. 1.47±0.23, n = 6, NS).

## Discussion

We have used a genetic engineering approach to convert unexcitable myofibroblasts from neonatal rat hearts into excitable cells with the objective of restoring rapid impulse propagation in a heterocellular monolayer model. The most important results are as follows: **1.** We generated excitable myofibroblasts by adenoviral overexpression of three ion channels: Kir2.1, Na_V_1.5, and Cx43. **2.** In patch clamp experiments, excitable NRVF demonstrated strong inward rectifier K^+^ currents that resulted in well polarized RMP near the predicted K^+^ equilibrium potential of −85 mV. These cells were readily excited by electrical stimuli similar to those used in NRVM in patch clamp experiments. When plated as 2D monolayers, these engineered excitable NRVF can propagate APs at velocities similar to those of NRVM. **3.** In heterocellular (NRVF/NRVM) monolayers, engineered excitable NRVF rescued CV to a level similar to pure myocyte monolayers. **4.** During reentry, both the complexity of wave propagation and the number of wavebreaks were significantly less in heterocellular monolayers containing excitable NRVF than non-excitable ones. Altogether, the results support the concept that cell therapy would benefit from using electrically excitable donor cells to increase safety of wave propagation and reduce arrhythmogenic potential.

### Gene/Cell Therapy for Cardiac Arrhythmias

Despite varying success of conventional therapies (pharmacology, ablation, and electronic devices) for cardiac arrhythmias, in many laboratories cell therapy has become a recent focus as a powerful tool to modify impulse initiation, conduction, or repolarization of the host myocardium [21]. Recent studies showed that human mesenchymal stem cells (hMSCs) overexpressing HCN2 channels can serve as biological pacemakers when they form gap-junctional electrotonic coupling with host cardiomyocytes [22]. Injection of these hMSCs into left ventricular wall successfully induced spontaneous rhythms in a chronic canine model of atrio-ventricular block [3]. Another recently developed technique utilized polyethylene glycol to induce cell fusion and create heterokaryons of donor and host cells. By using cell fusion the authors avoided uncontrolled migration and the need of high degree gap junction coupling to obtain a functional effect. Heterokaryons of host myocytes and HCN1-overexpressing myofibroblasts have been shown to generate spontaneous AP oscillations in guinea pig hearts [2]. These *in vivo* heterokaryons were suggested to remain stable in the host heart for several months [23], [24]. Human embryonic stem cells (hESCs) have also been efficacious in modulating heart rate. hESCs can differentiate into embryoid bodies that couple with neonatal rat myocytes and serve as biological pacemakers [25]. However, the *in vivo* effects are still controversial. Importantly, while most cellular therapies have been aimed at modulating pacemaking, very few have been developed for rescuing impulse propagation across diseased or scar tissue.

### Choice of NRVF as Candidate for Cell Therapy

To date the only published genetic modifications aimed at converting unexcitable cells into fully excitable have been in HEK293 cells. In 2005, Cho et al. attempted to generate pacemaker cells from HEK293 cells by expressing three ion channels: Kir2.1, NaChBac (a Na^+^ channel from bacteria), and human ether-a-go-go (hERG)-related channel. APs were generated in a small number of these cells (5/31) with low maximum rate of rise of 21.6±8.6 V/s [21]. More recently in 2011, Kirkton et al. generated a stable line of excitable HEK293 cells by modifying the overexpressed ion channel combination and delivering Kir2.1, Na_V_1.5, and Cx43 proteins into them [26]. In a subsequent study, the same group showed that excitable HEK293 cells improved both the electrical and mechanical function of a “zig-zag” network of cardiac tissues *in vitro* [27]. However, to our knowledge there is no previously published evidence of similar genetic modifications in unexcitable cells of cardiac origin.

We chose myofibroblasts in our study because they can form gap junctions with neonatal myocytes *in vitro* and directly modify the electrotonic properties of the myocytes [7], [28], [29]. Moreover, a recent cell therapy study has shown that engrafted myofibroblasts can integrate with host cardiomyocytes after transplantation in both rats and pigs [5]. In our study we utilized three adenoviral constructs to deliver ion channels that are essential for AP generation and propagation: Kir2.1, Na_V_1.5 and Cx43.

### Why Kir2.1, Na_V_1.5 and Cx43 Overexpression in NRVF?

Excitable NRVF generated APs whose morphology was reminiscent of APs in mammalian ventricular myocytes. However, in all cases the APD measured at 80% repolarization was relatively brief. Only during early stage of Ba^2+^ superfusion was APD_80_ prolonged to about 200 ms, just before complete Ba^2+^-induced depolarization occurred (Figure 4D). During the first few minutes of Ba^2+^ superfusion, the excitable NRVF were slightly depolarized and demonstrated a prominent plateau, which likely represented the time course of I_Na_ inactivation in the presence of strong inward rectification of I_K1_ [16]. Nevertheless, the question still arises as whether the endogenous sarcolemmal ion channels in NRVF contributed to the AP morphology demonstrated under our experimental conditions. Three endogenous voltage-gated potassium currents have been identified and characterized in NRVF [18]. The distribution of these potassium channels is very heterogeneous, but the transient outward K^+^ current (I_to_) expresses in the majority of cells; two different delayed rectifier K^+^ currents (I_Kf_ and I_Ks_) express in a small percentage of the cells; 4-AP was found to strongly block I_Kf_ and I_Ks_, but had no effect on I_to_. As demonstrated here, 4-AP blockade had negligible effects on the APD of excitable NRVF, which suggested that neither I_Kf_ nor I_Ks_ was involved. However, it is still possible that I_to_ contributes somewhat to the early phase of repolarization, but it is unlikely to have any role in maintaining the highly polarized resting membrane potential.

Walsh et al [18] also identified a small tetrodotoxin-sensitive Na^+^ current in some cells. We observed that in the absence of Na_V_1.5 overexpression, the small endogenous Na^+^ current was unable to depolarize the cells beyond threshold for AP initiation despite Kir2.1 overexpression, and a RMP close to −80 mV (Figure 4A). In a separate study, Na^+^-Ca^2+^ exchanger mRNA and proteins were identified, which may play a role in restoring the Na^+^ gradient in our engineered NRVF [30].

With regards to intercellular communication, studies have suggested that NRVF express gap junction proteins (Cx43, Cx45 and Cx40) across a number of species [7], [31], [32]. However, our immunostaining results indicated that the expression of either Cx43 or Cx40 was very limited in control NRVF (See Figure S5). Therefore, although Ad-Kir2.1+ Ad-Na_V_1.5 infection made single NRVF excitable, we were unable to demonstrate AP propagation in monolayers consisting of excitable NRVF expressing only endogenous connexins. However, excitable NRVF monolayers that overexpressed Cx43 demonstrated wave propagation at velocities that was comparable to those of NRVM. Therefore, the results suggest that in the setting of cell therapy, a sufficient density of gap junctional current would be necessary to enable AP propagation between excitable donor cells and the host myocardium. We also considered the possibility of using Ad-Kir2.1+Ad-Cx43, or Ad-Na_V_1.5+Ad-Cx43 as a means to rescue impulse propagation. On the one hand, treatment with Ad-Kir2.1+Ad-Cx43 would hyperpolarize NRVF. Thus, when coupled to neighboring cardiomyocytes, these cells would not be expected to alter the RMP of the cardiomyocytes. Yet they would surely increase the overall membrane capacitance without increasing the depolarizing inward current density. Consequently excitability would be reduced, leading to pro-arrhythmia. On the other hand, Ad-Na_V_1.5+Ad-Cx43 infected NRVFs will remain depolarized. Coupling these cells to the myocytes may reduce the RMP to an extent that no functional Na^+^ channels would be available. Therefore impulse conduction would also be impaired.

Our results significantly extended those of McSpadden et al [6], who conducted a study on cell pairs consisting of a NRVM coupled to an engineered unexcitable HEK293 cell of varying size expressing Kir2.1+Cx43. They demonstrated that pairing a large unexcitable HEK293 cell (e.g., ∼30 pF) to a NRVM reduced the myocyte RMP and distinctly impaired its ability to generate APs. On the other hand, small HEK293 cell size and the presence of I_K1_ contributed to the preservation of the RMP, as well as the excitability of the myocyte and the normal shape of its AP. The myofibroblasts used in the present study (∼82 pF at passage 3, Table 1) were larger than the HEK cells (∼31 pF) and also were significantly larger than the NRVM (∼12 pF). Yet the presence of Na_V_1.5 in those myofibroblasts not only helped preserve the ability of the myocytes to generate APs but it did so with a high degree of safety, as demonstrated by the significantly faster conduction velocity of heterocellular monolayers containing excitable myofibroblasts compared with monolayers containing unexcitable myofibroblasts. Thus, we can infer that, in the setting of cell therapy, the safety of wave propagation and prevention of pro-arrhythmia can only be guaranteed by the ability of both donor cells and host myocardium to generate and propagate rapid upstroke APs.

**Table 1:** Cell size of NRVF and NRVM.

	Range (pF)	Mean ± SEM (pF)
**Myofibroblasts**	27.83 to 240.6	82.31±8.85 (n = 33)
**Myocytes**	5.85 to 31.12	12.08±0.98 (n = 30)

### NRVF as a Target to Regulate Impulse Propagation

Several laboratories have developed a number of *in vitro* models to study the interaction of NRVF and myocytes and their role in modifying impulse conduction and arrhythmia [7], [29]. Rohr’s group significantly advanced our understanding in the consequences of heterocellular coupling using their patterned strands. Gaudesius et al. provided strong evidence that NRVF inserted between strands of NRVM can lead to significant conduction delay, and even conduction block when the length of NRVF exceeded 300 µm [29]. Subsequently, using a different heterocellular arrangement in their co-cultures, Miragoli et al. proved that the incidence of ectopic activity was in direct relationship with the density of NRVF cultured on top of myocytes-strands [7]. Experimental and simulation results from our laboratory demonstrated that in heterocellular monolayers containing myofibroblasts and myocytes distributed at random, the conduction velocity exhibits a complex nonlinear dependence on the degree of electrical coupling [8]. All of the above emphasizes the potential importance of the myofibroblasts as a target to regulate normal and abnormal impulse propagation and arrhythmogenesis in the fibrotic myocardium *in vitro*.

Recently, Rosker et al. tested the ability of three individual drugs to revert the phenotype of myofibroblasts to fibroblasts in cultured conditions [33]. They reported that 24 hour-treatment with an actin-targeting drug abolished the NRVF’s arrhythmogenic interaction with cardiomyocytes by increasing the CV in a dose-dependent manner. More recently, Jayawardena et al. demonstrated their ability to reprogram fibroblasts to cardiomyocytes using a combination of four micro-RNAs *in vitro* and *in vivo* [34], which further emphasized the importance of NRVF as a therapeutic target to regulate cardiac electrical activity. In our study, by genetically modifying NRVF using a combination of genes coding for three distinct membrane proteins that are essential in the initiation and propagation of cardiac electrical impulses we successfully improved conduction and reduced the arrhythmogenic effects of NRVF in an *in vitro* model.

### Study Limitations

The engineered excitable NRVF were generated and examined in an *in-vitro* neonatal rat model. While this allowed us to overexpress multiple ion channels in a highly controlled environment, the amount of virus required to make excitable NRVF could be different in large animals or humans because of the different expression of ion channels. Also we used uninfected NRVF instead of GFP infected NRVF in the co-culture system. In addition, adult myofibroblasts could be a better resource to further confirm our findings. Moreover, the adenoviral constructs of Kir2.1 used to increase I_K1_ was from mouse while the other two constructs were from human. While this would be problematic for *in vivo* testing, our goal was to demonstrate feasibility in an *in vitro* system. Nevertheless, our data suggested that the excitable NRVF can rescue impulse propagation and reduce the incidence of complex arrhythmias in heterocellular monolayers formed by myocytes and myofibroblasts.

Finally, we are well aware of the fact that introducing additional myofibroblasts into the atria or ventricles may be risky since they would increase extracellular matrix generation, and also secret a number of potentially damaging cytokines that might up- or down-regulate ion channel expressions in the host myocardium. Moreover, the viral constructs we used in this study are not specific for NRVF. Therefore, before injecting viral vectors and cells into injured myocardium, the safety of expressing these ion channels in both myocytes and NRVF *in vivo* needs to be tested. On the other hand other authors have recommended the use of adeno-associated viral gene delivery for long-term gene expression and adenoviral gene delivery for short-term proof-of-concept work [35]. Thus in regards to the clinical relevance of our study, we can only speculate as to the consequences of wholesale transduction of viral vectors into the damaged myocardium. In theory, we would see significant benefit in the overexpression of appropriate levels of the three most important genes controlling ventricular excitability and cell-to-cell propagation; i.e., Kir2.1, Na_V_1.5 and Cx43. The newly delivered genes would not only increase the excitability and conduction properties of the damaged myocytes themselves but also myofibroblast-myocyte coupling exist *in vivo*, then the transfer of these genes into the myofibroblasts might provide additional benefit to excitability in the injured zone and also contribute to prevent arrhythmias.

### Conclusion

Genetically modified NRVF are capable of generating and propagating APs similar to those generated by NRVM. Moreover, excitable myofibroblasts can couple with host cardiac myocytes and reduce arrhythmia by restoring rapid conduction and reducing fibrillatory conduction. Altogether, the data presented in this study strongly suggest that, in the setting of cell therapy, safety of wave propagation and prevention of pro-arrhythmia can only be guaranteed by the ability of both donor cells and host myocardium to generate rapid upstroke action potentials and propagate them at high velocities.

## Supporting Information

Figure S1 **Immunostaining of NRVF.** Immunostaining with α-smooth muscle actin (α-SMA, red) and DAPI (blue) showed that all cells in culture are myofibroblasts. These data are representative of 18 experiments.
(TIF)

Figure S2 **Characterizations of cell properties.** **A.** Cell size of neonatal rat ventricular myofibroblasts and myocytes. **B.** Cell capacitance of control cardiac myofibroblasts and triple infected myofibroblasts. **C.** Membrane resistance of control cardiac myofibroblasts and triple infected myofibroblasts. *: p<0.05.
(TIF)

Figure S3 **Exponential curve fit from FRAP experiments.**
(TIF)

Figure S4 **No signs of damage/necrosis in NRVF after infection. A.** Uninfected NRVF. **B.** Triple infected NRVF.
(TIF)

Figure S5 **Gap junction protein expression in control cardiac myofibroblasts when co-cultured with neonatal rat ventricular myocytes.** **A.** Cx43 expression between myofibroblasts and myocytes. **B.** Cx40 expression between myofibroblasts and myocytes.
(TIF)
