# Supplementary figures and images for: Genetically Engineered Excitable Cardiac Myofibroblasts Coupled to Cardiomyocytes Rescue Normal Propagation and Reduce Arrhythmia Complexity in Heterocellular Monolayers
Source: PLoS One. 2013 Feb 5;8(2):e55400. doi: 10.1371/journal.pone.0055400 (PMC3564921; doi:10.1371/journal.pone.0055400)

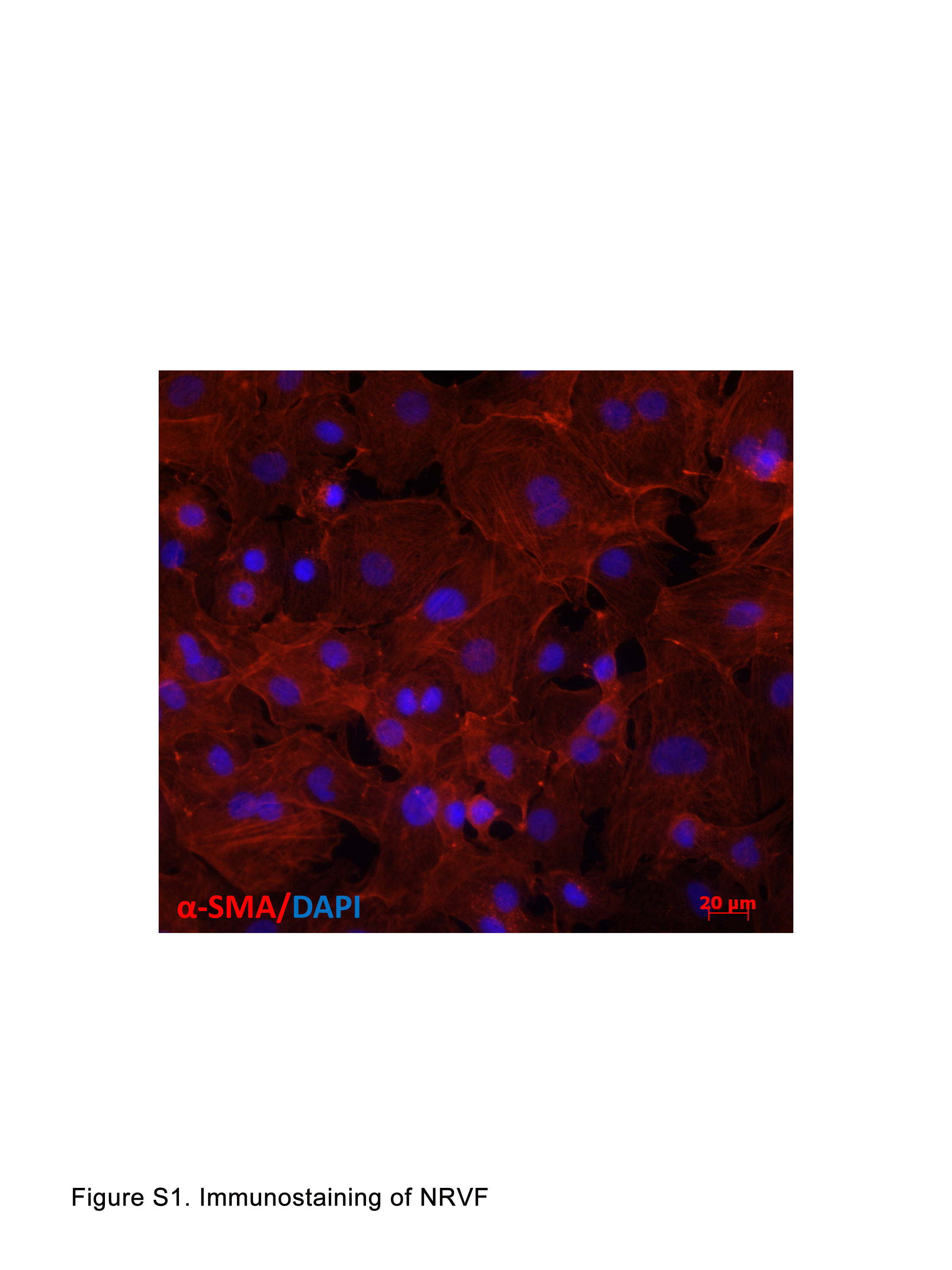

Supplement: Figure S1 — Immunostaining of NRVF. Immunostaining with α-smooth muscle actin (α-SMA, red) and DAPI (blue) showed that all cells in culture are myofibroblasts. These data are representative of 18 experiments. (TIF) [file pone.0055400.s001.tif]

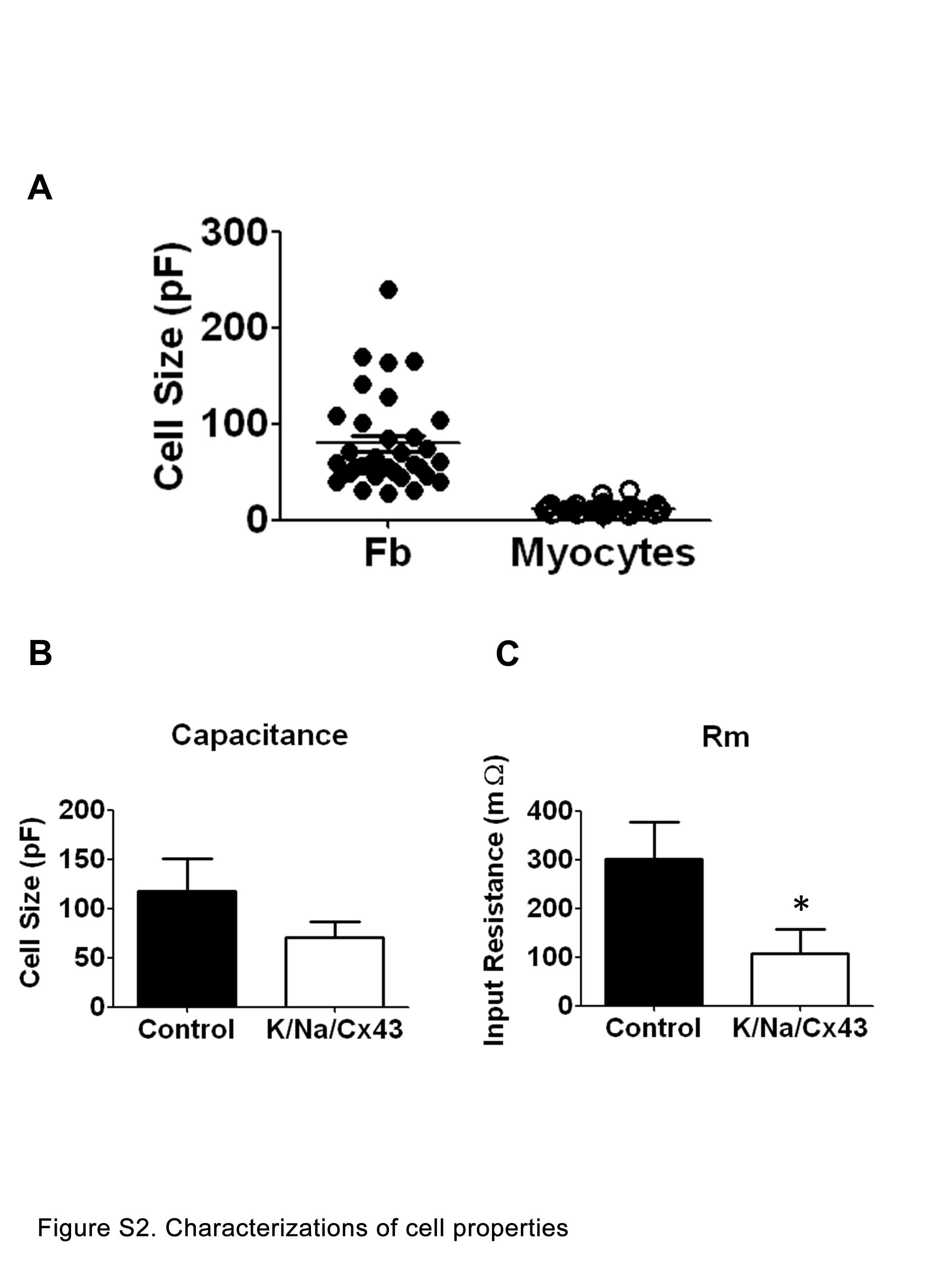

Supplement: Figure S2 — Characterizations of cell properties. A. Cell size of neonatal rat ventricular myofibroblasts and myocytes. B. Cell capacitance of control cardiac myofibroblasts and triple infected myofibroblasts. C. Membrane resistance of control cardiac myofibroblasts and triple infected myofibroblasts. *: p<0.05. (TIF) [file pone.0055400.s002.tif]

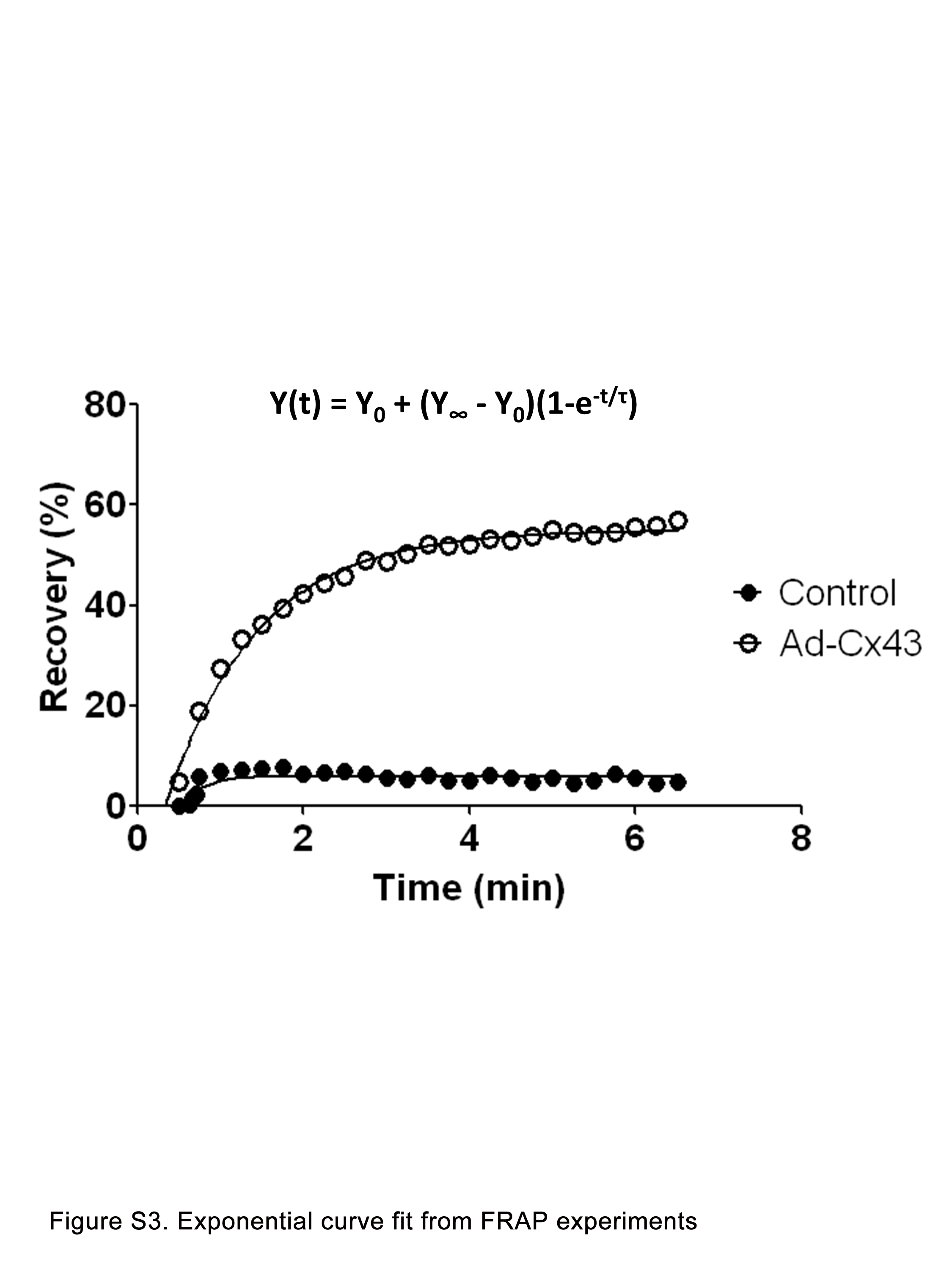

Supplement: Figure S3 — Exponential curve fit from FRAP experiments. (TIF) [file pone.0055400.s003.tif]

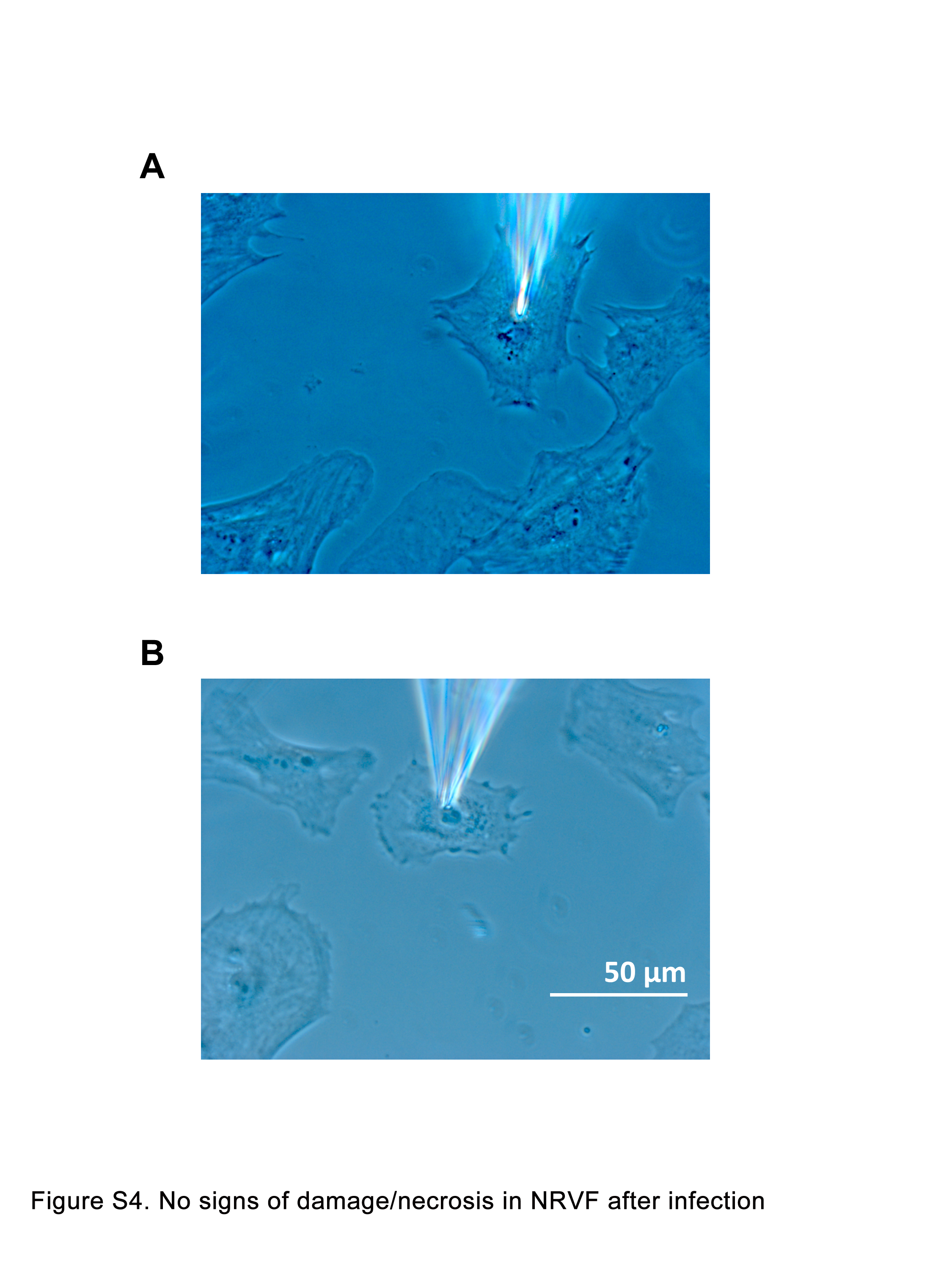

Supplement: Figure S4 — No signs of damage/necrosis in NRVF after infection. A. Uninfected NRVF. B. Triple infected NRVF. (TIF) [file pone.0055400.s004.tif]

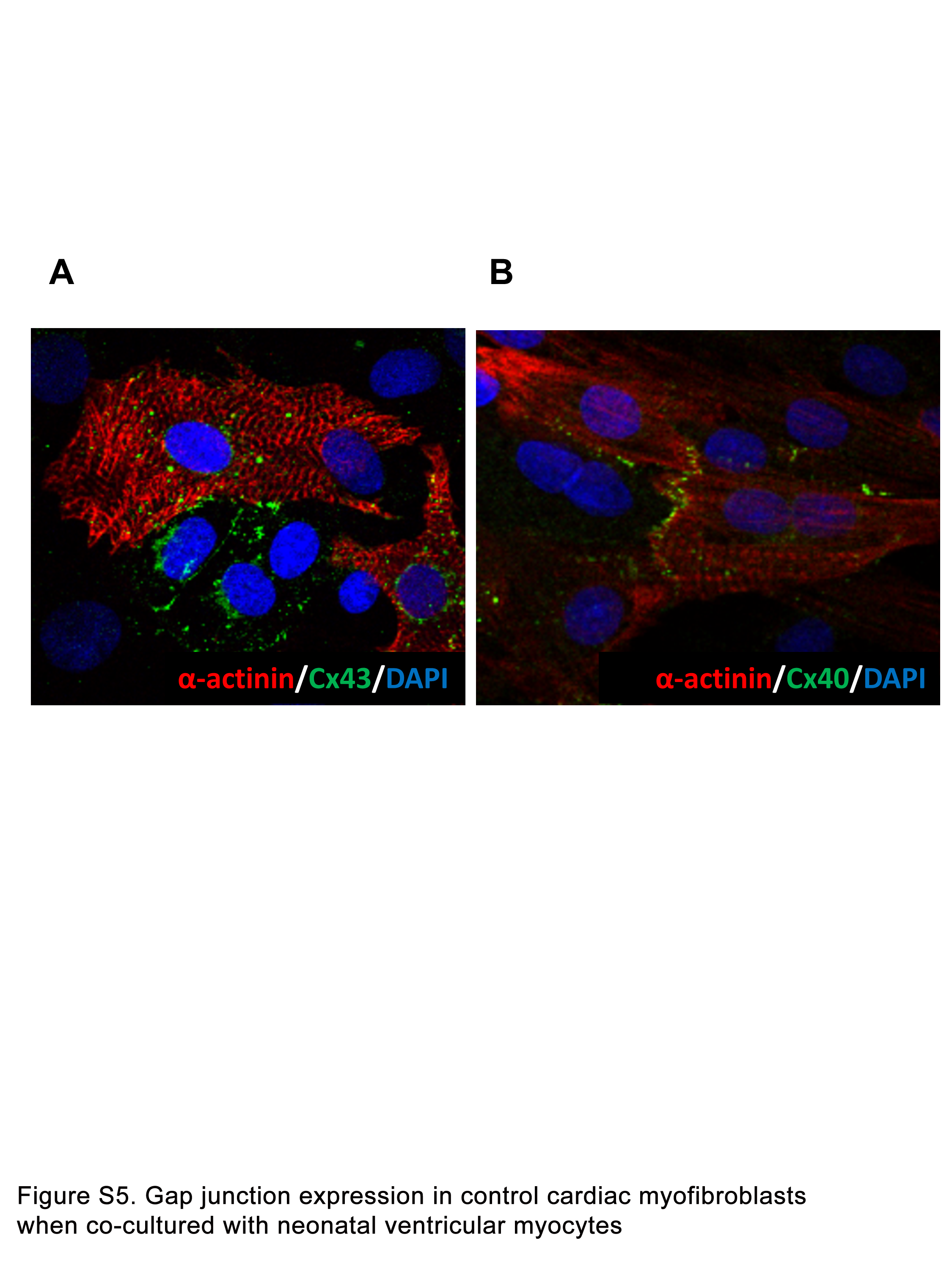

Supplement: Figure S5 — Gap junction protein expression in control cardiac myofibroblasts when co-cultured with neonatal rat ventricular myocytes. A. Cx43 expression between myofibroblasts and myocytes. B. Cx40 expression between myofibroblasts and myocytes. (TIF) [file pone.0055400.s005.tif]
